# Detection of Catalase as a major protein target of the lipid peroxidation product 4-HNE and the lack of its genetic association as a risk factor in SLE

**DOI:** 10.1186/1471-2350-9-62

**Published:** 2008-07-07

**Authors:** Anil D'souza, Biji T Kurien, Rosalie Rodgers, Jaideep Shenoi, Sadamu Kurono, Hiroyuki Matsumoto, Kenneth Hensley, Swapan K Nath, R Hal Scofield

**Affiliations:** 1 Department of Arthritis and Immunology, Oklahoma Medical Research Foundation, Oklahoma City, Oklahoma, USA; 2 College of Medicine, University of Oklahoma Health Sciences Center, Oklahoma City, Oklahoma, USA; 3 Iberica Co., Ltd., Kurume University Translational Research Center, Kurume-shi, Fukuoka 830-0011, Japan; 4 Dept. of Veterans Affairs Medical Center, Oklahoma City, Oklahoma, USA

## Abstract

**Background:**

Systemic lupus erythematosus (SLE) is a multifactorial disorder characterized by the presence of autoantibodies. We and others have implicated free radical mediated peroxidative damage in the pathogenesis of SLE. Since harmful free radical products are formed during this oxidative process, including 4-hydroxy 2-nonenol (4-HNE) and malondialdehyde (MDA), we hypothesized that specific HNE-protein adducts would be present in SLE red blood cell (RBC) membranes. Catalase is located on chromosome 11p13 where linkage analysis has revealed a marker in the same region of the genome among families with thrombocytopenia, a clinical manifestation associated with severe lupus in SLE affected pedigrees. Moreover, SLE afflicts African-Americans three times more frequently than their European-American counterparts. Hence we investigated the effects of a genetic polymorphism of catalase on risk and severity of SLE in 48 pedigrees with African American ancestry.

**Methods:**

Tryptic digestion followed by matrix assisted laser desorption/ionization time-of-flight mass spectrometry (MALDI-TOFMS) analysis was used to identify the protein modified by HNE, following Coomassie staining to visualize the bands on the acrylamide gels. Genotyping analysis for the C → T, -262 bp polymorphism in the promoter region of catalase was performed by PCR-RFLP and direct PCR-sequencing. We used a "pedigree disequilibrium test" for the family based association analysis, implemented in the PDT program to analyze the genotyping results.

**Results:**

We found two proteins to be HNE-modified, migrating around 80 and 50 kD respectively. Tryptic digestion followed by matrix assisted laser desorption/ionization time-of-flight mass spectrometry (MALDI-TOFMS) analysis of the Coomassie stained 80 kD band revealed that the target of HNE modification was catalase, a protein shown to associate with RBC membrane proteins. All the test statistics carried out on the genotyping analysis for the C → T, -262 bp polymorphism in the promoter region of catalase were non-significant (p > 0.05) in our data, which suggested that this SNP is not associated with SLE.

**Conclusion:**

Our results indicate that catalase is one of the proteins modified due to oxidative stress. However, catalase may not be a susceptibility gene for SLE. Nonetheless, catalase is oxidatively modified among SLE patients. This suggests a possible role between oxidative modification of catalase and its affects on enzymatic activity in SLE. An oxidatively modified catalase could be one of the reasons for lower enzymatic activity among SLE subjects, which in turn could favor the accumulation of deleterious hydrogen peroxide. Furthermore, HNE-products are potential neoantigens and could be involved in the pathogenesis of SLE. Decrease in catalase activity could affect the oxidant-antioxidant balance. Chronic disturbance of this balance in patients with SLE may work favorably for the premature onset of atherogenesis with severe vascular effect.

## Background

SLE is a complex, multisystem disorder with an unknown etiology and is distinguished by antibodies to self-proteins [1]. It virtually involves any organ system with a wide range of disease severity. 50–100% of SLE patients complained of fatigue, weight loss or fever in the absence of infection. The skin is affected in about three-fourths of patients, in the form of butterfly rash, photosensitivity rash, mucous membrane lesion, alopecia, Raynaud's phenomenon, purpura, urticaria or vasculitis. Two-thirds have musculoskeletal problems (arthritis, myositis or arthralgia). Renal problems are found in 16–38% of patients (proteinuria, haematuria, cellular casts or nephritic syndrome) [2]. Thirty-six per cent suffer from haematological problems (thrombocytopenia, anaemia or leucopenia) [3,4]. Reticuloendothelial anomaly is seen in 7–23% of patients (splenomegaly, lymphadenopathy or hepatomegaly). Neuropsychiatric problems are seen in 12–21% of patients (seizures, psychosis, transverse myelitis, brain syndrome, cranial neuropathies, and peripheral neuropathies); 18% suffer from gastrointestinal problems (vomiting, nausea or abdominal pain). Pulmonary problems are seen in 2–12% of the affected (pulmonary hypertension, pleurisy or pulmonary parenchymal disease [5]. Cardiac abnormalities are seen in 15% of patients (endocarditis, pericarditis or myocarditis).

Our earlier data showed oxidative damage occurring in SLE with significantly elevated conjugated diene and malondialdehyde formation [6]. Since a variety of harmful fragmentation products are formed during the oxidative process, including 4-HNE, which react with proteins to form potentially dangerous protein adducts [7], we hypothesized that specific HNE-protein adducts would be present in SLE. We investigated RBC membranes from 8 SLE and 11 normal subjects for HNE-modified proteins. We found two proteins to be HNE-modified, migrating around 80 and 50 kD, respectively. Tryptic digestion followed by MALDI-TOFMS analysis of the Coomassie stained 80 kD band revealed that the target of HNE modification was catalase, a protein shown to associate with RBC membrane proteins. RBC lysate catalase was not found to be modified, showing specificity of modification. We studied only the 80 kD band since this was found to be the most prominently affected in the SLE patients.

Catalase is an important endogenous antioxidant enzyme that detoxifies hydrogen peroxide to oxygen and water, thus limiting the deleterious effects of highly reactive oxygen species (ROS) [8]. Hence, catalase has been considered an important regulator of oxidative stress wherein chronic exposure to ROS may contribute to the development of SLE [9]. Catalase is located on chromosome 11p13 [10] where genetic linkage has been found among African-American SLE families with thrombocytopenia, a clinical manifestation associated with severe lupus in SLE affected pedigrees [11]. Moreover, African-Americans are three times more likely to be affected by SLE than European Americans, manifest SLE at an earlier age, and have a clinically more severe phenotype than other American racial groups [12]; however, there have been no genetic studies that have directly focused on the contribution of the catalase gene to SLE in African Americans. Because of its location within the 11p13 genetic linkage for SLE, we investigated a genetic polymorphism of the catalase gene [C(-262)T (rs1001179)], on the risk and severity of SLE in 48 pedigrees with African-American ethnic background and found no genetic associations.

## Methods

### Materials

Anti-4-HNE keyhole limpet hemocyanin was obtained from Biotrend Chemikalien, Cologne, Germany. Anti-rabbit IgG alkaline phosphatase conjugate was from Jackson Laboratories, Bar Harbor, ME. ECL plus chemiluminescence kit was from Amersham Biosciences, Piscataway, NJ. Bicinchoninic acid (BCA) protein assay kit was from Pierce Chemical Company, Rockford, IL. Pre-cast sodium dodecyl sulfate polyacrylamide gel electrophoresis (SDS PAGE) gels (4–20% gradient gel) were from ISC Bioexpress, Kaysville, UT. HotStart DNA polymerase kit was purchased from Qiagen. All primers were synthesized by Molecular Biology Resource facility at OMRF. All other reagents used were of reagent grade.

### Human sera and study participants

Blood was drawn under a protocol approved by the Institutional Review Board. All patients satisfied the 1982 American Rheumatism Association revised criteria for the classification of SLE [13]. SLE patients and their family members were enrolled in the lupus genetics studies previously described [14]. Enrollment of a family required at least 2 members who fulfill the 1982 revised or modified criteria for SLE, as previously reported and whose relationship was informative for linkage with an SLE phenotype. All patient material was gathered under protocols approved by the Oklahoma Medical Research Foundation and University of Oklahoma Health Sciences Center Institutional Review Boards and after informed consent was obtained from participants. Clinical manifestations were defined as either present or absent based on the definitions in the SLE classification criteria. Sera was serologically evaluated for ANA, anti-double-stranded (ds) DNA, anti-Sm, antiphospholipid immunoglobulin G (IgG) and IgM antibodies using specimens obtained for this project by following standard procedures. Anti-Ro, anti-La, anti-P, and anti-nRNP (nuclear ribonucleoprotein) were also evaluated by using Ouchterlony immunodiffusion in each affected subject. Evidence for biologic false-positive test for syphilis and the lupus anticoagulant was extracted from the medical record. These clinical evaluations are performed routinely as part of patient and sample recruitment (11).

### Purification of red blood cell (RBC) membranes and preparation of membrane ghost

Red blood cells were purified from the peripheral blood of eight SLE patients and eleven age and sex matched controls. Membrane ghosts were prepared by hypotonic lysis [15].

### Detection of HNE-modified proteins in RBC membranes

The RBC membrane proteins were quantified using the Pierce's BCA Protein Assay kit using bovine serum albumin (BSA) as the standard. RBC samples were analyzed on pre-cast SDS PAGE [16] gels (4–20% gradient gel) (ISC Bioexpress, Kaysville, UT). Equal amounts of protein were loaded in all lanes. Gels were electrophoretically [17] transferred onto nitrocellulose membranes. Following blocking, the membranes were probed with rabbit anti-HNE antibodies (Biotrend Chemikalien, Cologne, Germany). The membranes were washed thrice with Tris-buffered saline, pH 7.4 (five minutes each) and then incubated with anti-rabbit IgG horseradish peroxidase conjugate for 1 hour. The wash was repeated and the HNE-modified proteins were visualized using ECL plus Western blotting system chemiluminescence kit (Amersham Biosciences, Piscataway, NJ).

Three RBC samples were chosen and a gel was run with two identical halves. After electrophoresis, the gel was cut in half. One half was immunoblotted; the other was stained with Coomassie blue and dried. The Coomassie stained band which migrated at 80 kD was excised from the gel after comparison with an identical anti-HNE-stained immunoblot and subjected to in-gel tryptic digestion. The tryptic fragments were analyzed using MALDI-TOFMS and a database search was used to identify the HNE-modified proteins [18].

### Catalase genotyping

**Figure 1:**
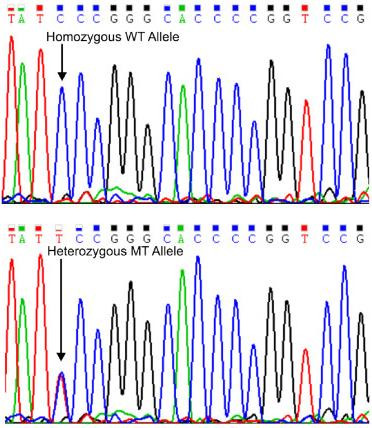
**Representative partial chromatograms: Sequencing of the PCR product were obtained using the following two primers; F: 5'-attccgtctgcaaaactggc-3' and R: 5'-gagcctcgccccgccggcccg-3'.** The top panel depicts the sequence from a normal homozygous allele and the bottom panel depicts the sequence form an affected individual. The arrows indicate the position of the base that is variable between the heterozygous sample (bottom panel), where both bases, T and C, are present on the two alleles and the wild type sample (top panel).

DNA was isolated from blood samples collected from 223 individuals (100 affected and 113 matching controls (also see table 1). Genotyping was conducted by restriction-fragment length polymorphism (RFLP)-PCR, without knowledge of subject status. Genotyping analysis for the C → T, -262 bp polymorphism in the promoter region of catalase was performed by PCR-RFLP and direct PCR-sequencing (Figure 1). Approximately 10 ng of DNA was amplified by thermal cycling using the HotStart DNA polymerase kit with PCR buffer containing 1.5 mM MgCl_2_, 0.2 mM of each dNTP, 0.5 U Taq, and 8 pmol of each primer. PCR conditions included an initial denaturation at 95°C for 15 min followed by 35 cycles of 94°C for 30 s, 60°C for 45 s, and 72°C for 30 s, with a final extension at 72°C for 10 min. Following restriction enzyme digestion (2 U), products were stained with ethidium bromide, resolved by 2% agarose gel electrophoresis and visualized using a FluorChem UV imaging system or in most cases sequenced directly.

**Table 1:** Catalase SNP (rs 1000179) in cases with SLE and normal controls and pedigree disequilibrium test

	SLE (cases)		Controls	
		
	Males	Females	Males	Females
Participants	9	102	28	84
C/C genotype	7	90	28	74
C/T genotype	2	12	0	10

	S1 (avg)	S2(sum)	S3(genotype)	

TD0 (both)	0.776	0.8575	0.8575	
TD1 (only sib pairs)	0.9263	1	1	
TD2 (only triads)	0.3173	0.3173	0.3173	

We have used 48 multigenerational, multiplex families for our family-based association analysis using the PDT program [19]. The test retains a key property of the "transmission disequilibrium test (TDT)", in that it is valid even when there is population substructure or stratification. The PDT program performs both allele-specific and genotype-specific LD analysis of individual markers. T-test was used to assess the significance of for mean differences in general characteristics between SLE and normal subjects. We did not find a single individual in our pedigrees that presented with a T/T genotype, though the reported T/T genotype in African American population is 3.2%.

**Figure 2:**
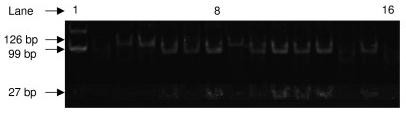
**Restriction Fragment Length Polymorphism Analysis:** The C/T polymorphism alters a Sma-I site in the SNP (rs1001179), therefore an antisense primer 5'-gagcctcgccccgccggcccg-3' that incorporates a mutation in order to abolish an exisiting Sma-I site was used together with sense primer 5'-attccgtctgcaaaactggc-3' which amplifies a 126 bp fragment. Hot Start PCR reactions were performed using 10 ng genomic DNA, without knowledge of sample status, using the following conditions; initial denaturation at 95°C for 15 min followed by 35 cycles of 94°C for 30 s, 60°C for 45 s, and 72°C for 30 s, with a final extension at 72°C for 10 min. The entire reaction was then digested with 2 U of Sma-I and analysed on 2% agarose gels after staining with ethidium bromide and visualization on a FluorChem UV imaging system. The T allele eliminates a SmaI restriction site. 99 bp and 27 bp fragments are visualized in the wild-type C allele (see lanes 6, 7, 8, 10, 11, 12, 13, and 14) while the full length PCR product are seen in lanes 2, 3, 4, 5 and 9.

The CAT -262 C → T promoter polymorphism was genotyped using the following primers: F: 5'-attccgtctgcaaaactggc-3' and R: 5'-gagcctcgccccgccggcccg-3'. The T allele eliminates a SmaI restriction site. The primers amplify a 126-bp fragment that is cut into 99 and 27 bp fragments in the wild-type C allele, while if the T allele is present SmaI does not cut resulting in the full length PCR product (Figure 2). Using a unique three base SNP assay developed in house, we further studied 4 additional SNP's (RS 1049982, RS 11546300, RS 3180560 and RS 769217) that span the catalase gene, including the 5' and 3' UTR regions.

### Statistical analysis

We have used 48 multigenerational, multiplex families for our family-based association analysis. Our data consists of larger pedigrees with multiple nuclear families and/or discordant sib-ships. Therefore, it was desirable to have a valid statistical test of association (linkage disequilibrium) that can use all potentially informative data, even from the extended pedigrees. To do that, we have used a "pedigree disequilibrium test" in our family based association analysis, implemented in the PDT program [19]. The test retains a key property of the "transmission disequilibrium test (TDT)", in that it is valid even when there is population substructure or stratification. The PDT program performs both allele-specific and genotype-specific linkage disequilibrium analysis of individual markers. T-test was used to assess the significance of mean differences in general characteristics between SLE and normal subjects.

## Results and Discussion

**Figure 3:**
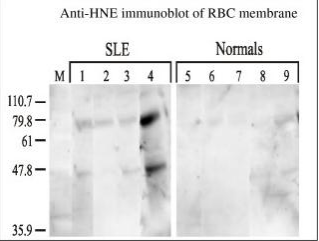
**Anti-4 hydroxy-2-nonenal immunoblot of RBC membrane of SLE and control samples: RBC membrane ghosts were prepared as mentioned earlier.** Equal amounts of protein samples were electrophoresed and transferred to nitrocellulose membrane and immunoblotted with anti-HNE antibodies. Anti-rabbit horseradish peroxidase conjugate was added and the blots were developed using chemiluminecence. Lanes 1–4 corresponds to RBC membranes obtained from SLE samples while lanes 5–9 correspond to normal controls. The samples were analyzed at random on the gel and later cut out and aligned after identification as either SLE or normals. 'M' stands for molecular weight standard.

**Figure 4:**
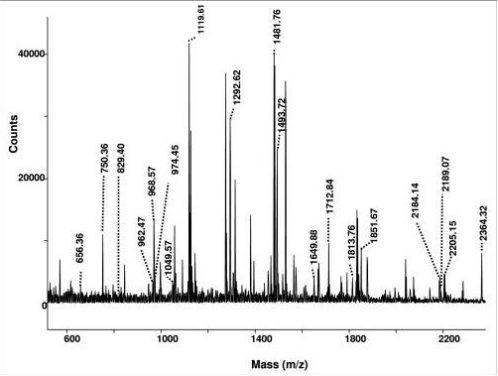
**The mass spectrum showing the tryptic fragments of the 80 kD band analyzed by MALDI-TOF-MS that matched to sequences from catalase.** The Coomassie stained band migrating at 80 kD was excised and subjected to in-gel tryptic digestion and MALDI-TOF-MS. The mass of each individual peak was used to perform a Mascot Search, which identified human catalase as the primary candidate antigen (38% match). Of the 59 mass values searched, 19 of them matched to sequences obtained from catalase. The probability based Mowse score obtained for the Mascot search analysis was 100, where a score of 64 is significant (p < 0.05). X-axis gives the mass to charge ratio (m/z) and Y-axis gives the intensity of the peaks in counts.

Our interest to see whether oxidative damage occurred in RBC membranes of SLE patients stemmed from our observation of increased oxidative damage in the sera of SLE patients compared to normals. Upon loading equal amounts of proteins in all lanes during electrophoresis, the anti-HNE antibodies recognized two proteins, migrating approximately at 80 and 50 kD. These proteins were not detected in the normal membrane samples above background (Figure 3). This showed that oxidative damage had occurred in the RBC membranes of SLE patients. The targets of HNE-modification were limited and similar between patients. In order to identify one of the modified proteins, the 80 kD band was excised out of SDS PAGE gels and subjected to tryptic digestion followed by MALDI-TOF MS. Nine out of ten times the results of mass spectrometric analysis (Figure 4) identified the target of modification as catalase. Results were inconclusive one time consequent to insufficient protein. It does appear that catalase may be susceptible to HNE-modification even in normal patients as there is some evidence of it (see lane 9), however, such modification is more intense and more common in the SLE patients.

RBC membranes are rich in polyunsaturated fatty acids and hence are potential targets for oxidative modification. Lipid peroxidation occurs as part of the oxygen toxicity process when increased oxidative stress disrupts the prooxidant/antioxidant balance. During this process free radicals mediate oxidative damage of polyunsaturated fatty acids. We were interested to identify proteins that were modified by HNE, the most studied form of the 4-hydroxy-2-alkenal group of α, β-unsaturated aldehydes. HNE has two very electrophilic sites, the alkene bond and the aldehyde group. The alkene bond reacts with three nucleophilic amino acids, cysteine, lysine, and histidine. The product of this reaction is thought to be the cyclic hemiacetal form that equilibriates with the open chain aldehyde form. The free aldehyde in the open chain enables it to react with a second amino acid and thus act as heterobifunctional crosslinking agents.

Catalase, a tetrameric antioxidant enzyme with a molecular weight of 59800, catalyses the hydrolysis of the deleterious hydrogen peroxide, formed by the action of superoxide dismutase (SOD) to water. In our results, we find a discrepancy in the migration of catalase, which migrates around 80 kD. This anomaly in electrophoretic migration is possibly a direct result of modification with HNE, with the additional HNE molecules resulting in a higher molecular weight. It may be also possible that the additional HNE molecules hinder migration. Studies have shown catalase to be cytosolic. However, previous data have shown catalase interacting with cell membrane proteins and also shown the enzyme to be found in membrane isolates [20].

The finding that catalase is a target of HNE-modification in SLE raises interesting questions. Catalase, part of the defense system against free radical damage, is itself a target. Though catalase has been affected, there is no widespread free radical damage, as might be expected. This may be due to the fact that cytosolic catalase appeared to be unaffected or due to the increased scavenging of hydrogen peroxide by glutathione peroxidase. However, several reports show that glutathione peroxidase activity is significantly lowered in SLE [21,22]. These studies also showed decreased levels of reduced glutathione and elevated levels of oxidized glutathione as well as decreased catalase and SOD activities. The decrease in catalase activity could be due to modified catalase bound to RBC membrane, which together with decreased glutathione peroxidase activity could favor the accumulation of hydrogen peroxide. This in the presence of ferrous ions leads to the more deleterious hydroxyl radicals through Fenton's reaction. Preliminary results show that there were no antibodies directed against catalase in the sera of SLE patients. However, we had observed antibodies against the extracellular SOD1. It would be interesting to see if there are antibodies against HNE-modified catalase, since HNE modification of the enzyme could enable it to behave as a neoantigen [23]. Recent data show that transgenic mice over-expressing human catalase localized to the mitochondria lived 4.5 months longer than the wild type controls [24], showing the importance of catalase in aging and how HNE-modification of this important antioxidant enzyme could be deleterious.

To test whether the catalase promoter single nucleotide polymorphism (SNP); rs1001179, was associated with SLE in such a way to explain our previous linkage at 11p13, we performed a family-based association analysis. Since we have multigenerational, multiplex families, we have used pedigree disequilibrium test (PDT). We have used 3 statistics, S1 (avePDT), S2 (SumPDT) and S3 (genoPDT) on all the families consisting of only sibpairs (TD1), only triads(TD2) or both (TD2) samples to assess the genetic association. All the test statistics were non-significant (p > 0.05) in our data (Table 1), which suggested that this SNP is not associated with SLE.

Previous results from our group [11,25] show sufficient evidence to establish genetic linkage at chromosome 11p13. A recent study also detected a marker on the same locus in families with discoid lupus erythematosus [26]. In addition, the SNP we investigated for this study (rs1001179), which results in a C/T substitution at position -262 in the catalase promoter region, has been shown to influence transcription factor binding and reporter gene transcription [27]. Furthermore, it has also been shown to be associated with increased blood levels of catalase. Catalase activity measured from serum samples of SLE patients have been shown to be significantly lower than normal controls [27]. Moreover, lipid peroxidation as measured by serum MDA concentration was significantly elevated in SLE patients and was positively correlated with the SLE activity index [28].

On further review of the hapmap around the region of 11p13 where catalase is found to be mapped, strikingly, catalase is found to be mapped within one single haploblock in the CEU, CHB and JPT populations. The only exception is the Yoruba tribe, wherein, the catalase gene is split into three haploblocks. This tribe most likely represents the African-Americans haploblock. On further review of the coding SNP's in the catalase gene, we found 5 SNPs, namely rs35677492, rs17880442, rs11032709, rs769217 and rs17886350, however, every single of these coding SNP's was a non-informative synonymous SNP. Using an unique 3 base SNP assay developed in house [29], we studied one of the above SNP's (rs769217) along with 3 others (rs1049982; found at the 5'-UTR region, rs11546300 and rs3180560; found at the 3'UTR region) and our results suggest a linkage in the region but no association of the catalase gene with SLE (results not shown). These results confirm our previously reported linkage in this region (11).

Catalase has also been implicated in another autoimmune and polygenic disease, viz. vitiligo [30]. We have correlated thrombocytopenia with the severity of SLE with a genetic marker located in the region of the genome that also contains catalase. The present results, despite the small number of patients, however, suggest that the genetic polymorphism of catalase does not play a significant role in the development of SLE in the African-American population, even though the frequency of the variant allele in our SLE samples is 3 times what NCBI reports for their 48 African American samples (0.126 vs. 0.042). Nonetheless, our results strongly implicate catalase to be one of the major protein targets of HNE-modification in the RBC membranes of SLE patients. Previously, we and others have shown increased oxidative damage in SLE [11].

## Conclusion

The current data suggests catalase to be one of the proteins modified due to this oxidative stress. Even though catalase may not be a susceptibility gene for SLE, nonetheless, catalase is oxidatively modified among SLE patients. This suggests a possible role between oxidative modification of catalase and its affects on enzymatic activity in SLE. An oxidatively modified catalase could be one of the reasons for lower enzymatic activity among SLE subjects, which in turn could favor the accumulation of deleterious hydrogen peroxide. Furthermore, HNE-products are potential neoantigens and could be involved in the pathogenesis of SLE. Decrease in catalase activity could affect the oxidant-antioxidant balance. Chronic disturbance of this balance in patients with SLE may work favorably for the premature onset of atherogenesis with severe vascular effect [20].

## Abbreviations

Systemic lupus erythematosus: SLE; red blood cell: RBC; 4-Hydroxy-nonenol: 4-HNE; matrix assisted laser desorption/ionization time-of-flight mass spectrometry: MALDI-TOFMS; reactive oxygen species: ROS; sodium dodecyl sulfate polyacrylamide gel electrophoresis: SDS PAGE; Bicinchoninic acid: BCA; bovine serum albumin: BSA; restriction-fragment length polymorphism: RFLP; superoxide dismutase: SOD; single nucleotide polymorphism: SNP; pedigree disequilibrium test: PDT; malondialdehyde: MDA.

## Competing interests

The authors declare that they have no competing interests.

## Authors' contributions

AD and BTK jointly wrote the manuscript and have provided equal contributions to this manuscript. AD carried out the RFLP analysis and designed the genotyping assays. BTK, designed the MALDITOF experiments. RR carried out the Western blots. JS carried out the red cell membrane preps, SK carried out the Mass Spectometry, HM analyzed and interpreted the Mass Spectrometry data, KH provided the Anti-HNE antibody and critically revised the manuscript. SKN provided the statistical analysis and RHS provided critical input during the preparation of this manuscript, been involved during the drafting of this manuscript and gave final approval for this manuscript to be published. None of the authors have any competing personal or financial interests'. All authors read and approved the final manuscript.

## Pre-publication history

The pre-publication history for this paper can be accessed here:


